# Molecular signaling of the HMGB1/RAGE axis contributes to cholesteatoma pathogenesis

**DOI:** 10.1007/s00109-014-1217-3

**Published:** 2014-11-12

**Authors:** Miroslaw J. Szczepanski, Michal Luczak, Ewa Olszewska, Marta Molinska-Glura, Mariola Zagor, Antoni Krzeski, Henryk Skarzynski, Jan Misiak, Karolina Dzaman, Mikolaj Bilusiak, Tomasz Kopec, Malgorzata Leszczynska, Henryk Witmanowski, Theresa L. Whiteside

**Affiliations:** 1 Department of Otorhinolaryngology, Faculty of Medicine and Dentistry, Medical University of Warsaw, Warsaw, Poland; 2 Department of Clinical Immunology, University of Medical Sciences, Poznan, Poland; 3 Department of Computer Science and Statistics, University of Medical Sciences, Poznan, Poland; 4 Department of Otolaryngology, University of Medical Sciences, Poznan, Poland; 5 Department of Otolaryngology, Medical University of Bialystok, Bialystok, Poland; 6 Department of Pathology and Laboratory Medicine, Brown University, Providence, RI 02912 USA; 7 Institute of Physiology and Pathology of Hearing, Warsaw, Poland; 8 Department of Physiology, University of Medical Sciences, Poznan, Poland; 9 Department of Pathology, University of Pittsburgh School of Medicine, University of Pittsburgh Cancer Institute, 5117 Centre Avenue, Pittsburgh, PA 15213 USA

**Keywords:** RAGE/HMGB1 axis, Keratinocytes, Cholesteatoma pathogenesis, Inflammation

## Abstract

**Abstract:**

Cholesteatoma represents progressive expansion of the keratinizing squamous epithelium in the middle ear with subsequent chronic inflammation in subepithelial connective tissues. The hypothesis was tested that receptor for advanced glycation endproduct (RAGE) and its ligand, high-mobility box 1 (HMGB1), are overexpressed in cholesteatoma, and the RAGE/HMGB1 axis might contribute to its pathogenesis. Cholesteatoma samples (*n* = 36) and 27 normal skin specimens were studied by immunohistochemistry (IHC) for HMGB1 and RAGE expression. Effects of HMGB1 signaling on proliferation, migration, cytokine production, and apoptosis of human immortalized keratinocytes (HaCaTs) and normal keratinocytes were studied by quantitative reverse transcription (qRT)-PCR, IHC, Western blots, and flow cytometry after cell co-incubation with HMGB1. While all studied tissues expressed HMGB1, its expression was higher in cholesteatoma than in normal skin (*p* < 0.0001). All cases of cholesteatoma also showed elevated RAGE expression levels, and only 7/27 (26 %) of normal skin specimens were weakly positive for RAGE. Proliferation and migration of HaCaT cells incubated with HMGB1 were up-regulated (*p* < 0.05). HMGB1 also prevented HaCaT cell apoptosis and induced activation of several molecular signaling pathways in keratinocytes. The data suggest that in cholesteatoma, HMGB1 released from stressed or necrotic epithelial cells and binding to RAGE overexpressed in keratinocytes initiates molecular signaling that culminates in pro-inflammatory cytokine release and chronic inflammation.

**Key message:**

- HMGB1 signaling engages multiple activation pathways in RAGE-positive keratinocytes.
- HMGB1 protects RAGE-positive keratinocytes from drug-induced apoptosis.
- Keratinocyte proliferation is controlled via RAGE and HMGB1 molecular signaling.
- Molecular signaling of the HMGB1/RAGE axis contributes to cholesteatoma pathogenesis.

**Electronic supplementary material:**

The online version of this article (doi:10.1007/s00109-014-1217-3) contains supplementary material, which is available to authorized users.

## Introduction

The immune system has evolved to respond not only to pathogens, but also to signals released from dying cells. Sensing the presence of pathogens provides an initial signal for the immune system to respond to the invader [1, 2]. This process requires the presence of pattern recognition receptors (PRRs) that detect pathogen-associated molecular patterns (PAMPs). The best studied of these receptors are Toll-like receptors (TLRs). The immune system is also able to recognize endogenous danger signals or damage-associated molecular patterns (DAMPs), which involve molecules released by necrotic or dying cells [3]. When cells die through necrosis, they induce several molecular pathways. In contrast, apoptotic cell death induces tolerance [4]. The DAMP superfamily includes various breakdown products of the extracellular matrix, e.g., the DNA-binding high-mobility box 1 (HMGB1) protein which has been widely studied [5]. HMGB1 plays a crucial role in molecular responses to cell damage/necrosis via activation of the nuclear factor kappa B (NF-κB) transcription factor and subsequent induction of pro-inflammatory cytokines, interleukin (IL)-1 and tumor necrosis factor alpha (TNF-α) [6]. The receptor for advanced glycation endproducts (RAGEs) is the major receptor for DAMPs [4]. RAGE activation via its multiple ligands, including HMGB1, plays a key role in various diseases, including acute or chronic inflammatory conditions such as sepsis and diseases such as rheumatoid arthritis, diabetic nephropathy, and cancer [6–9].

Cholesteatoma is a destructive disease characterized by the progressive expansion of keratinizing squamous epithelium in the middle ear and mastoid and chronic inflammatory reaction of the subepithelial connective tissue which is accompanied by bone destruction with intracranial extensions. Many theories of its pathogenesis have been proposed but failed to provide insights into the molecular background of disease, prevention, or cure. Cholesteatoma tissue is composed of proliferating, migrating, and highly keratinizing epithelium known as the matrix and of subepithelial connective tissue infiltrated by inflammatory cells, antigen-presenting cells, fibroblasts, and endothelial cells and known as the perimatrix [10, 11]. Cholesteatoma tissue is also the source of pro-inflammatory cytokines such as IL-1, transforming growth factor alpha (TGF-α), pro-angiogenic factors, and various enzymes [12–15]. It is suspected but not proven that this mix of soluble factors activates osteoclasts, which are engaged in bone destruction [10].

Here, we report for the first time the co-expression of HMGB1 and RAGE in cholesteatoma tissues and hypothesize that their molecular interaction resulting in activation of several major intracellular signaling pathways may be responsible for cellular hyperplasia, cell migration, and resistance to apoptosis characteristic of cholesteatoma. Although a Mongolian gerbil animal model of cholesteatoma has been described [16], concerns exist about its relevance to human disease [17]. Since we have not been able to establish an in vivo cholesteatoma model, we have resorted to a series of molecular in vitro studies with human keratinocytes to investigate the potential role of HMGB1 in the cholesteatoma pathogenesis. The results of our ex vivo studies were correlated with the expression in human cholesteatoma tissues of the proteins shown to be involved in keratinocyte activation and proliferation. Based on our ex vivo and in situ studies, we propose that high RAGE expression levels in cholesteatoma tissues might be the key factor contributing to the disease progression.

## Materials and methods

### Patients

Thirty-six tissue samples were obtained from 15 females and 21 males diagnosed with acquired middle ear cholesteatoma who underwent surgery for the first time. The patients’ ages ranged from 17 to 78 years (median = 48). The presence of pathologically documented middle ear cholesteatoma in surgically removed tissues was required for entry into the study. Tissues were fixed in 10 % formaldehyde, and sections stained with hematoxylin–eosin (H + E) were prepared and evaluated by light microscopy. Control tissues included noninflammatory skin specimens from the external auditory canal (*n* = 9) or from the retroauricular area (*n* = 18). Seven fresh middle ear cholesteatoma tissue samples and seven control tissue samples were used for protein extraction. The study was approved by the Local Ethics Committees at the Poznan University of Medical Sciences (#290/04 to MJS) and at the Medical University of Bialystok (#R-I-002/89/2010 and #R-I-002/125/2011 to EO) and was conducted as recommended by the Helsinki Declaration.

### Cell cultures

Normal human keratinocyte cell line human immortalized keratinocyte (HaCaT) and Normal Adult Human Primary Epidermal Keratinocytes (ATCC® PCS200011™) were purchased from CLS Cell Lines Service GmbH (Eppelheim, Germany) and from ATCC, respectively. HaCaT cells were maintained in RPMI-1640 medium (Sigma-Aldrich Chemie GmbH, Steinheim, Germany) containing 10 % (*v*/*v*) heat-inactivated fetal calf serum (FCS), 2 mM glutamine, 100 mg/mL streptomycin, and 100 U/mL penicillin. Normal Adult Human Primary Epidermal Keratinocytes were maintained in serum-free Dermal Cell Basal Medium (ATCC® PCS200030) supplemented with components of the keratinocyte growth kit (ATCC® PCS200040) that contains the following growth supplements: bovine pituitary extract (BPE), rhTGF-α, l-glutamine, hydrocortisone hemisuccinate, insulin, epinephrine, and apotransferrin. Both cell lines were grown at 37 °C in a 5 % CO_2_ humidified atmosphere. Cells in confluence were harvested, washed twice with phosphate-buffered saline (PBS), and detached from culture flasks by a brief treatment with 0.02 % EDTA solution (Sigma-Aldrich Chemie GmbH).

### Reverse transcription and real-time quantitative PCR analysis of receptor for advanced glycation endproduct and Toll-like receptor 4 transcripts

The primers used for RAGE amplification were F = 5′-GAAGGGAGTATCTGTGAAGGAA-3′ and R = 5′-AGCTACAGGAGAAGGTGGGA-3′; for TLR4 amplification, F = 5′-ACAACCTCCCCTTCTCAACCA-3′ and R = 5′-GTGGCTTAGGCTCTGATATGC-3′; and for PBGD (a housekeeping gene) amplification, F = 5′-GCCAAGGACCAGGACATC-3′ and R = 5′-TCAGGTACAGTTGCCCATC-3′. The quantitative real-time reverse transcription (RT)-PCR was performed as previously described [18].

### Flow cytometry

A Becton Dickinson flow cytometer equipped with FACSDiva v6.1.2 software was used to evaluate surface RAGE expression in cells. To evaluate intracellular HMGB1 expression, cells were permeabilized with saponin (0.1 % in PBS) for 20 min, washed, and incubated with anti-HMGB1 antibody (Ab). At least 2 × 10^4^ HaCaT cells were acquired for analysis. The following Abs were used for staining: polyclonal rabbit anti-human HMGB1 (Abcam, Cambridge, USA), polyclonal rabbit anti-human RAGE (LifeSpan Bioscience Inc., Seattle, WA, USA), and secondary Ab FITC-labeled donkey anti-rabbit IgG (Jackson ImmunoResearch Laboratories, Inc., West Grove, USA). Before staining, all Abs, including isotype control Ab, were pretitrated to establish optimal staining dilutions, and the staining was performed as previously described [18].

### Immunohistochemistry

The following primary Abs were used for immunostaining of tissue sections: rabbit polyclonal anti-human: HMGB1 (Abcam), RAGE (LifeSpan Bioscience Inc.), phosphorylated Akt (pAkt), pc-Jun, pMAPKp44/p42, pMEK1/2, pSTAT3, pNF-κβ p65 (all from Cell Signaling Technology, Danvers, MA, USA), and isotype control IgG (Dako, Gdynia, Poland). Paraffin sections of normal skin, cholesteatoma, and head and neck squamous cell cancer (HNSCC) tissues were handled as previously described [19, 20]. As a positive control, HNSCC tissues were stained for Akt (pAkt), pc-Jun, pMAPKp44/p42, pMEK1/2, pSTAT3, and pNF-κβ p65. After standard de-paraffinization, the EnVision+ System (Dako) was used for staining according to the manufacturer’s instructions. Slides were evaluated in a light microscope (at ×200 or ×400 magnification) or in an inverted Olympus FluoView 10 laser scanning confocal microscope under an oil immersion objective. For digital image analysis, the AnalySIS^B software was used. All stained sections were analyzed and scored by two independent investigators (M.J.S. and M.L.) to avoid bias, and the two scores were averaged and recorded. The sections were scored according to the % of cholesteatoma tissue staining (positivity <25 % = 0, 25–75 % = 50, and >75 % = 100). The level of staining intensity was recorded as none = 0, weak = 1, moderate = 2, or strong = 3. Using these values, the H score was calculated for each sample by multiplying positivity by intensity.

### Silencing of Toll-like receptor 4 expression with lentivirus particles

HMGB1 is a ligand for RAGE; however, it also activates TLR4, and therefore, to avoid co-signaling, TLR4 was silenced in HaCaT cells using five small hairpin RNA (shRNA) Lentiviral Clones (Sigma-Aldrich Chemie GmbH) targeting the NM_003266 sequence. The TLR4 gene KO was preformed according to the manufacturer’s protocol as previously described by Pi et al. [21]. The TRC1.5 pLKO.1-puro Empty Vector Control Transduction Particles (Sigma-Aldrich Chemie GmbH) and TRC1.5 pLKO.1-puro-CMV-TurboGFP Positive Control Transduction Particles (Sigma-Aldrich Chemie GmbH) were used as a missense control and positive control for measuring transduction efficiency and optimizing shRNA delivery, respectively.

### Treatment of cell lines with high-mobility box 1

Cells were seeded in wells of 6- or 24-well plates (5 × 10^5^/mL). HMGB1 (Sigma-Aldrich) was added at the concentrations of 10–200 ng/mL, and cells were incubated for various time periods. Cells were also cultured in medium alone or without FCS (controls). In all experiments, HaCaT cells permanently silenced for TLR4 with lentivirus particles (Sigma-Aldrich) were used. For functional assays, plates were incubated at 37 °C for 6 to 96 h. Supernatants were collected and stored frozen at −20 °C for cytokine analyses. Each sample was evaluated in triplicate.

### Blocking of high-mobility box 1 effects

In some experiments, anti-RAGE blocking Abs or isotype control Abs (both from R&D Systems Inc., Minneapolis, MN, USA) were used to determine whether interference with HMGB1 signaling inhibits cell proliferation and migration. In preliminary titration experiments, the Ab concentration of 10 μg/mL was found to be able to almost completely block HMGB1-mediated effects.

### SDS-PAGE and Western blots for signaling pathway components in human immortalized keratinocyte cells

The Abs used for immunostaining of phosphoproteins were also used for Western blots. Rabbit polyclonal anti-GAPDH (FL-335) and goat anti-rabbit IgG HRP-conjugated Abs were purchased (Santa Cruz Biotechnology, Santa Cruz, CA, USA). Western blots were performed as previously described [18] using RIPA buffer (Sigma-Aldrich Chemie GmbH) supplemented with Halt Protease and Phosphatase Inhibitors (Thermo Scientific, Rockford, IL, USA) and PMSF (Sigma-Aldrich Chemie GmbH). The Western blots were quantified using ImageJ 1.46r software (National Institutes of Health, USA).

### Cell proliferation and migration assays

Cell proliferation and migration assays were performed using the xCELLigence System (Roche Diagnostics GmbH) with RTCA DP Instrument (Roche Diagnostics GmbH) according to the manufacturer’s protocol. When the cells entered the logarithmic growth phase, culture medium was aspirated and replaced with fresh culture medium ± HMGB1 at the concentration of 10–200 ng/ml. Cell proliferation and migration were continuously monitored every 5 min using the RTCA DP Instrument via calculation of the “cell index” (to reflect the surface area covered by the cells) for each plate. Each sample was evaluated in triplicate.

### Measurements of cytokines in cell culture supernatants

The quantitative determinations of IL-1β and TNF-α levels in supernatants were performed using a Cytometric Bead Array Flex Set (BD Biosciences) and analyzed by a FACSCanto flow cytometer (BD Biosciences) equipped with Fax Diva Software, according to the manufacturer’s instructions. IL-8 was detected in supernatants from HaCaT cultures using a human IL-8 Quantikine ELISA kit (R&D Systems) used as per the manufacturers’ instructions.

### Annexin V binding assays

Apoptosis after treatment of HaCaT cells with HMGB1 followed by incubation with cisplatin was analyzed by flow cytometry, and Annexin V binding to cells was measured using an Annexin V kit purchased from Beckman Coulter as previously described [20].

### NF-κB and STAT3 translocation and signaling molecule phosphorylation

To evaluate NF-κB or STAT3 expression and Akt, c-Jun, MAPK, and MEK1/2 activation, the translocation to nucleus of the NF-κB p65 subunit and of STAT3 and pAkt, c-Jun, MAPK, and MEK1/2 expression were measured in an Olympus FluoView 10 confocal microscope using HaCaT cells stained with rabbit: anti-human NF-κB p65 (Santa Cruz Biotechnology) and anti-human STAT3 (Cell Signaling Technology) Abs and Abs specific for pAkt, c-Jun, MAPKs, and MEK1/2 as previously described [20].

### Statistical analysis

Data were summarized by descriptive statistics. Fisher’s exact tests were used to determine if there was a difference in RAGE and HMGB1 expression among the tissue types. Adjustments to *p* values were made using the Bonferroni step-down procedure. The paired Student’s *t* test was used to evaluate differences between treated vs. untreated pairs of cell lines. The *P* value of <0.05 was considered to be significant.

## Results

### RAGE and HMGB1 expression in cells and tissues

**Fig. 1:**
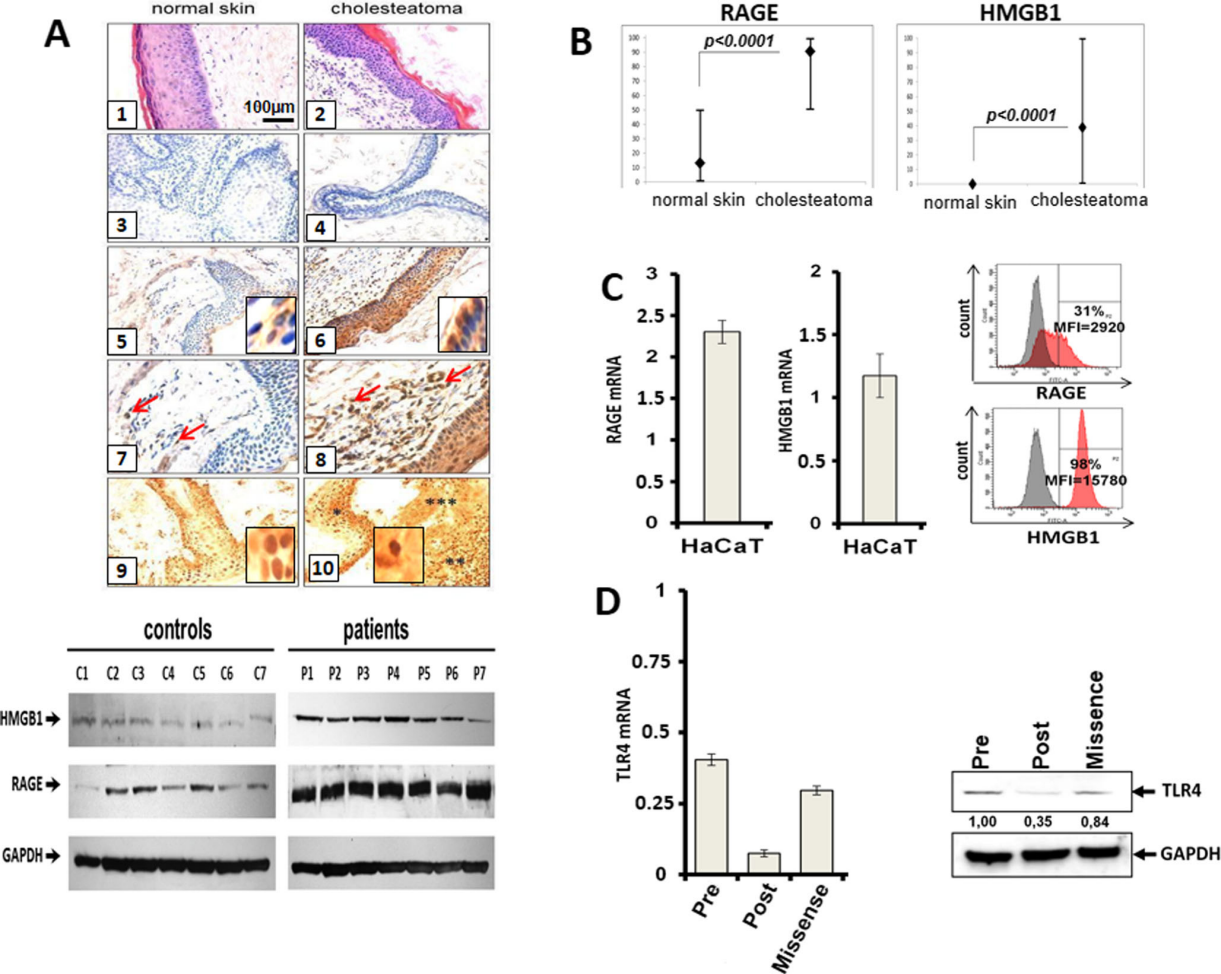
RAGE, HMGB1, and TLR4 expression in normal skin, cholesteatoma, and the cell line. **a** (*1*) normal skin of external auditory canal, H + E staining (×200); (*2*) cholesteatoma tissue, H + E staining (×200); (*3*) isotype control in normal skin (×200); (*4*) isotype control in the cholesteatoma (×200); (*5*) cytoplasmic RAGE expression in normal skin epithelium (×200), *inset* (×600); (*6*) cytoplasmic RAGE expression in the matrix of cholesteatoma (×200), *inset* (×600); (*7*) RAGE expression in dermis layer of the skin, single positive cells are present (*arrows*) (×400); (*8*) RAGE expression in the perimatrix of cholesteatoma, numerous positive cells are present (*arrows*) (×400); (*9*) nuclear and cytoplasmic HMGB1 expression in normal skin epithelium (×200), *inset* (×600); and (*10*) nuclear, cytoplasmic, and extracellular HMGB1 expression in cholesteatoma matrix (*), in perimatrix (**), and on debris released from necrotic cells (***) (×200), *inset* (×600). **b** Expression of RAGE and HMGB1 in normal skin vs. cholesteatoma. The sections were scored according to the % and level of staining intensity of the tissues. The H score was calculated for each sample as described in “Materials and methods.” The H scores for RAGE and HMGB1 expression in normal skin epithelium vs. cholesteatoma tissue are compared. **c** RAGE and HMGB1 expression at the mRNA and protein levels was determined in HaCaT cells by RT-PCR (*left*) and flow cytometry (*right*), respectively. The *gray peaks* indicate mean fluorescence intensity (*MFI*) of isotype control, while the *red peaks* show MFI of RAGE or HMGB1. **d** TLR4 mRNA and protein levels in HaCaT cells prior to and post-stable silencing with the lentiviral vector or silencing with scrambled RNA (“missense”)

The presence of epithelium in paraffin sections of normal skin and cholesteatoma tissues was the requirement for evaluating RAGE and HMGB1 expression. Using immunohistochemistry and Western blot techniques, we found significant differences in RAGE expression levels between cholesteatoma epithelium and normal skin epithelium (*p* < 0.0001) (Fig. 1a). In cholesteatoma tissues, RAGE was detectable in the cytoplasm and in cell nuclei (Fig. 1a (6)). In normal skin, RAGE expression was absent in 74 % of specimens or weak in 26 % (Fig. 1a (5)). RAGE expression in the normal skin epithelium was confined to the basal layers (23/27 = 85 %) or to the basal/granular layers (4/27 = 15 %). We also found that RAGE was expressed in sebaceous glands of normal skin, where its staining intensity ranged from moderate to strong (data not shown). RAGE was expressed in all cases of cholesteatoma, and RAGE staining intensity ranged from moderate to strong (Fig. 1a (6)). In 31 patients (86 %), the staining intensity was strong, and in 5 (14 %), it was moderate. In cholesteatoma, all epithelial layers were RAGE positive. In the dermis, RAGE expression was confined to single fibroblasts or immune cells (Fig. 1a (7)). In the cholesteatoma perimatrix, a large number of inflammatory cells were evident, and these cells (Fig. 1a (8)) as well as endothelial cells (not shown) were strongly RAGE positive.

We also observed significant differences in HMGB1 expression between normal skin and cholesteatoma tissues (*p* < 0.0001). In normal skin, HMGB1 was expressed in all viable nucleated cells (Fig. 1a (9)) and was not expressed extracellularly. In cholesteatoma tissues, HMGB1 was also expressed in all viable and nucleated cells in the matrix and perimatrix. However, abundant extracellular accumulations of HMGB1 were also found in the debris released from necrotic cells (Fig. 1a (10)). Figure 1b summarizes the differences in RAGE and HMGB1 expression between normal skin epithelium and cholesteatoma. No differences in RAGE and HMGB1 expression vs. clinical advance or localization of cholesteatoma in the middle ear were found.

**Fig. 2:**
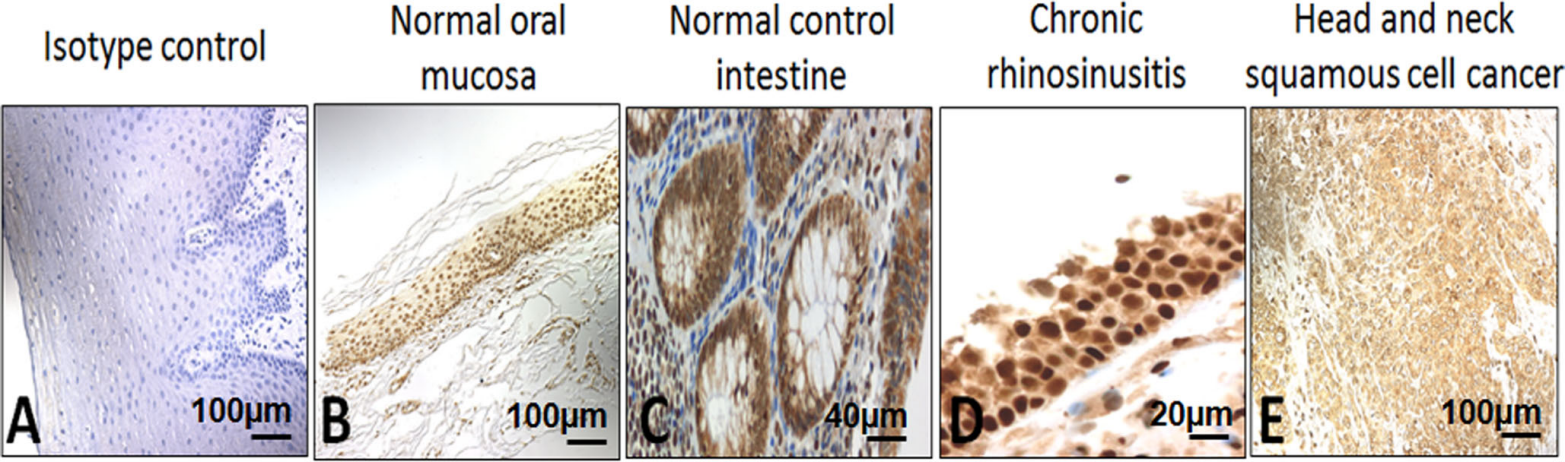
HMGB1 expression in normal tissues, chronic inflammation, and cancer tissues. **a** Isotype control in normal oral mucosa (×100). **b** Normal oral mucosa. **c** Normal control intestine. **d** Chronic rhinosinusitis with nasal polyps (×200). **e** Head and neck squamous cell cancer (×100)

In preliminary experiments, immunohistochemistry (IHC) for HMGB1 was performed using a variety of normal human tissues rich in inflammatory infiltrates such as oral mucosa, intestine, or nasal polyps of patients with chronic rhinosinusitis and also tumor issues such as HNSCC. HMGB1 was found to be expressed in all of these tissues (Fig. 2).

Using quantitative reverse transcription (qRT)-PCR and flow cytometry, messenger RNA (mRNA) and protein for RAGE and HMGB1 were found to be expressed in HaCaT cells (Fig. 1c) and in Normal Adult Human Primary Epidermal Keratinocytes (data not shown). In fact, the levels of expression for RAGE and HMGB1 were comparable in both lines.

By qRT-PCR and Western blots, mRNA and protein levels of TLR4 were measured before and post-stable TLR4 gene silencing in HaCaT cells. TLR4 expression was decreased by 80 % in HaCaT cells after gene silencing.

### High-mobility box 1 promotes molecular signaling, leading to proliferation, migration, and interleukin-8 secretion by receptor for advanced glycation endproduct-positive keratinocytes

**Fig. 3:**
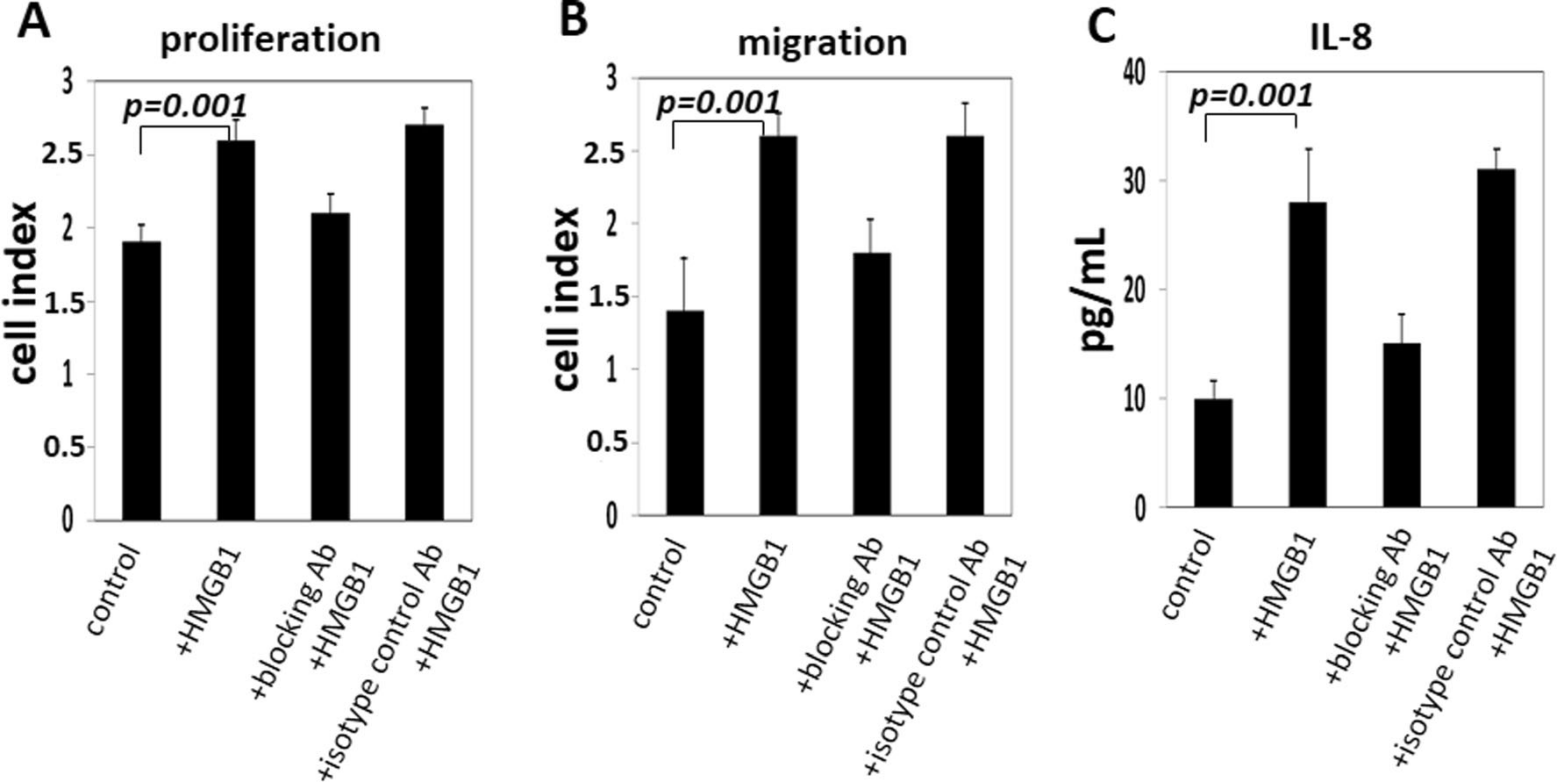
The effect of HMGB1 on proliferation, migration, and cytokine production by TLR4-silenced HaCaT cells. Cell proliferation and migration assays were performed after 72 h of culture using the xCELLigence System, and IL-8 level was measured by ELISA assay, as described in “Materials and methods.” HMGB1 (100 ng/mL) induced significant proliferation, migration, and IL-8 production in HaCaT cells. Antibodies specific for RAGE almost completely blocked the effects mediated by HMGB1. The data are from six experiments with each sample run in triplicate, and the *p* values confirm significance of the receptor blockade

Effects of HMGB1 on cell proliferation were studied in the cell lines, which were either exposed to HMGB1 at the concentration of 100 ng/mL or pretreated with blocking anti-RAGE Abs. As shown in Fig. 3a, b, HMGB1 significantly enhanced proliferation of TLR4-silenced HaCaT cells (*p* = 0.001) and increased their migration (*p* = 0.001). The effects of HMGB1 on the cell lines were almost completely abrogated (*p* = 0.001) in HaCaT cells in the presence of RAGE-specific blocking Abs. These blocking experiments were repeated using different concentrations of anti-RAGE antibodies, and they conclusively demonstrated that HMGB1/RAGE signaling was responsible for the observed effects. Increased production of IL-8 in supernatants of HaCaT cells cultured with HMGB1 was observed, and this effect was also abrogated (*p* = 0.001) in the presence of RAGE-specific blocking Abs (Fig. 3c). We did not detect other inflammatory cytokines, e.g., TNF-α or IL-1β in HaCaT supernatants, either before or after their co-incubation with HMGB1.

### High-mobility box 1 protects receptor for advanced glycation endproduct-positive keratinocytes from drug-induced apoptosis

**Fig. 4:**
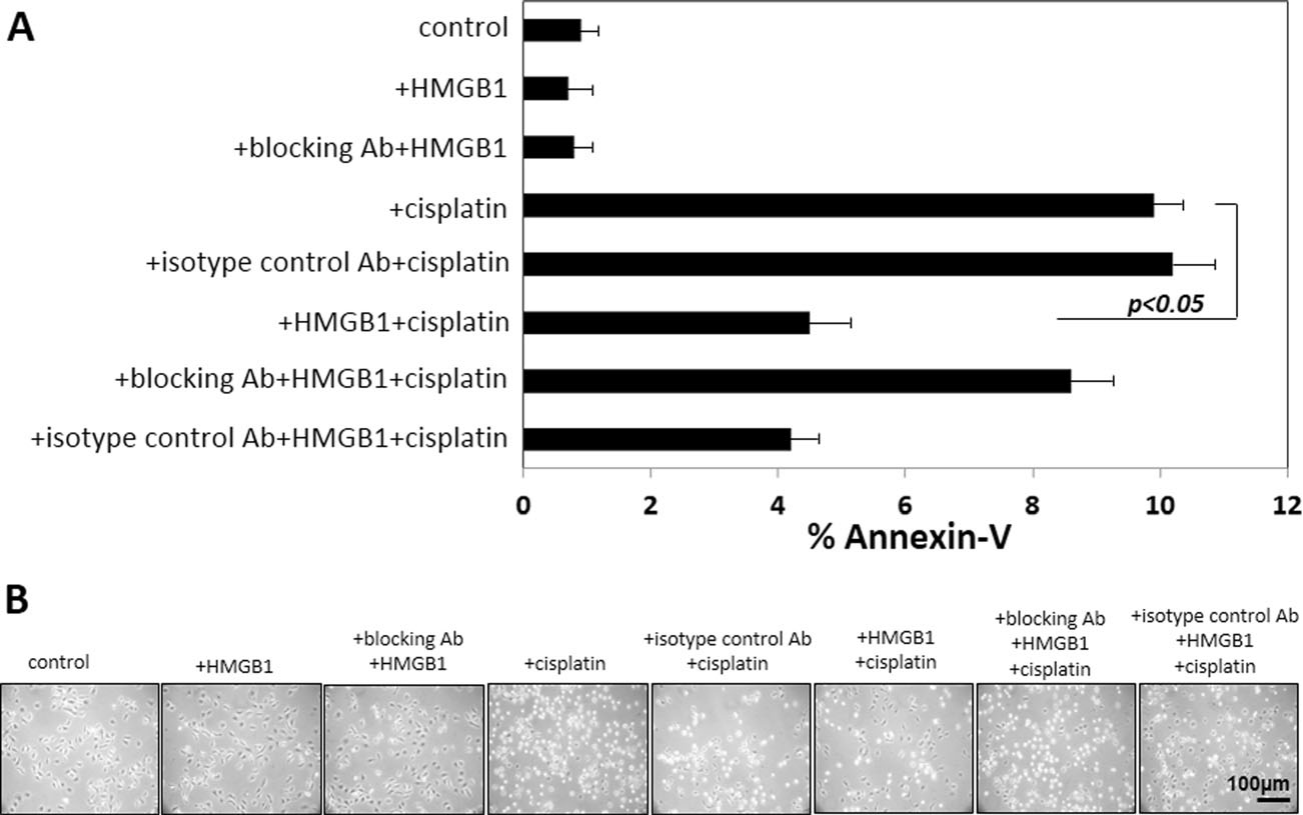
Effects of HMGB1 pretreatment on cisplatin-induced apoptosis in TLR4-silenced HaCaT cells. HaCaT cells were treated with HMGB1(100 μg/mL). **a** HMGB1 pretreatment protects cells from apoptosis induced by cisplatin. Cell lines were first incubated ± HMGB1 for 24 h and then with cisplatin (10 μmol/L) for 6 h, stained with Annexin V and propidium iodide, and examined by flow cytometry. **b** Morphologic changes in HaCaT cells incubated ± cisplatin or + HMGB1 and cisplatin. Magnification ×200. Results are representative of six independent experiments

After stimulation with HMGB1 alone, apoptosis levels remained unchanged in TLR4-silenced HaCaT cells as measured by Annexin V binding. Apoptosis was decreased (*p* < 0.05) when HaCaT cells were pretreated with HMGB1 before treatment with cisplatin (Fig. 4a), HaCaT cells treated with cisplatin as previously described [20] showed an altered morphology, with many cells undergoing apoptosis (Fig. 4b). However, the effect of the drug was partially abrogated when tumor cells were preincubated with HMGB1 (100 ng/mL). The data suggest that HMGB1 protects RAGE-positive cells from cisplatin-induced apoptosis.

### High-mobility box 1 signaling engages multiple activation pathways in receptor for advanced glycation endproduct-positive keratinocytes

**Fig. 5:**
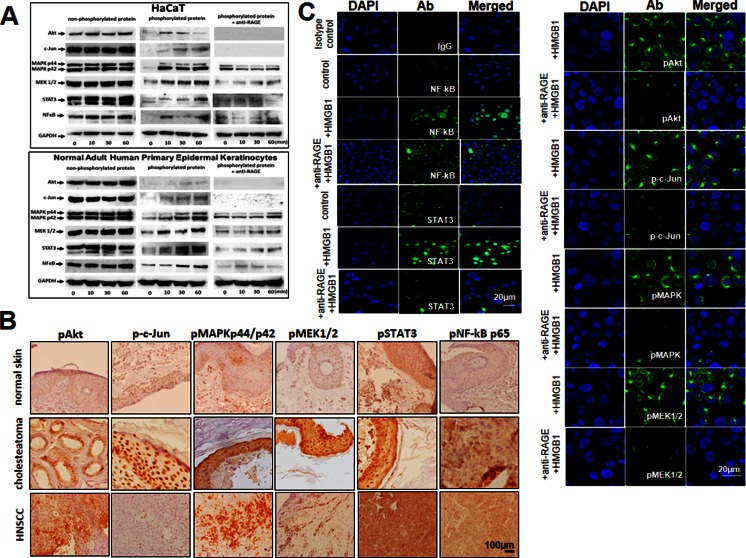
Expression of signaling molecules in TLR4-silenced HaCaT cells and in tissue sections and translocation of NF-κB p65 and STAT3 upon triggering with HMGB1. **a** Western blots of HaCaT and normal adult human primary epidermal keratinocytes incubated ± HMGB1 show phosphorylation of the PI3K/Akt, c-Jun, MAPKp44/p42, MEK1/2, STAT3, and NF-κB. **b** Increased levels of all phosphorylated signaling molecules in cholesteatoma tissue vs. normal skin. HNSCC tissues (the *lowest row*) were used as a positive control (×200). **c** HaCaT cells plated overnight were stimulated with HMGB1 (100 ng/mL) ± anti-RAGE Ab for 12 h and then stained using isotype control IgG or antibodies for NF-κB and pSTAT3, pAkt, pc-Jun, pMAPK, and pMEK1/2, as described in “Materials and methods.” Control cells were unstimulated. *Bar* = 20 μm. Results are representative of three independent experiments

To evaluate molecular pathways engaged in the induction of HaCaT cell proliferation, migration, and resistance/sensitivity to drug-induced apoptosis upon stimulation with HMGB1, components of the PI3K/Akt survival pathway as well as c-Jun, MAPKp44/p42, MEK1/2, STAT3, and the NF-κB pathway components were examined. All these molecules were constitutively expressed in the nonphosphorylated form in TLR4-silenced HaCaT cells and in normal adult human primary epidermal keratinocytes (Fig. 5a). The phosphorylated forms of these proteins were either not expressed or only weakly expressed in HaCaT cells not stimulated with HMGB1 (Fig. 5a). After HMGB1 stimulation for 10 to 60 min, phosphoprotein levels dramatically increased in HaCaT cells (Fig. 5a), and this effect was partially inhibited in the presence of anti-RAGE blocking Abs (data not shown). As this Ab was shown to interfere with HMGB1-mediated induction of proliferation, migration, and survival of RAGE-positive HaCaT cells (see above), our data suggest that HMGB1-mediated effects are mediated through phosphorylation of these proteins.

Importantly, the results obtained with cell lines corresponded to the expression of signaling phosphoproteins in tissues. We found that all cholesteatoma tissues expressed the above-described phosphoproteins. Further, their expression levels ranged from moderate to strong (Fig. 5b). In contrast, expression levels of these phosphoproteins were undetectable or weak in normal skin (Fig. 5b), except for the pSTAT3 protein which showed moderate expression level in epithelial cells. This level of expression corresponded to the baseline expression in HaCaT cells unstimulated with HMGB1 (Fig. 5a).

### High-mobility box 1 induces translocation of nuclear factor kappa B p65 and STAT3 transcription factors to the nucleus

Next, translocation of a p65 subunit of NF-κB or pSTAT3 to the cell nucleus was evaluated after stimulation of TLR1-silenced HaCaT cells with HMGB1 (Fig. 5c). The baseline translocation levels for p65 subunit and pSTAT3 were first established in HaCaT cells and found to occur in 15 ± 7 and 9 ± 3 % (mean ± SD) of cells, respectively. After treatment with HMGB1, the p65 subunit translocation occurred in 49 ± 12 % of the cells (*p* < 0.05) and the pSTAT3 translocation in 40 ± 15 % of the cells (Fig. 5c). These effects were partially but significantly (*p* < 0.01) blocked by anti-RAGE Abs. These results further indicate that NF-κB and STAT3 pathways mediate the HMGB1 effects in RAGE-positive cells.

## Discussion

Emerging data indicate that chronic ear inflammation is associated with the cholesteatoma development and progression. We have reported that TLR2, TLR3, and TLR4 are expressed in epithelial cells, including human acquired cholesteatoma and cancer cells [19, 20, 22]. It is also clear that cholesteatoma growing in a space-limited area of the middle ear can exceed the capacity of the existing vessel supply. This might result in molecular changes such as apoptosis [23] or hypoxia and necrotic cell death within the cholesteatoma tissue, followed by release of HMGB1. Most likely, it is HMGB1 which triggers paracrine activation of RAGE expressed in the cholesteatoma matrix but not (or only weakly) in normal skin.

In this study, we demonstrated for the first time co-expression of RAGE and its endogenous ligand, HMGB1, in cholesteatoma. Their co-expression was significantly up-regulated in cholesteatoma compared to normal skin epithelium. Of special interest is the finding of extracellular accumulations of HMGB1 in cholesteatoma tissues, suggesting that release of HMGB1 from cells and its abundance contribute to chronic inflammation. In the microenvironment of middle ear cholesteatoma, RAGE/HMGB1 signaling might be, at least in part, responsible for molecular mechanisms that contribute to the development of the inflammatory disease.

To examine the molecular signaling pathways potentially involved in cholesteatoma induction, we used an in vitro HaCaT model. The molecular signaling in this cell line does not necessarily explain the pathogenesis of cholesteatoma. For this reason, responses of keratinocytes to HMGB1-induced signaling were compared to signals and expression patterns present in vivo, in the cholesteatoma tissues. In the absence of an in vivo animal model of cholesteatoma, this approach allowed us, in part, to reconstruct the cellular and molecular events that are likely to be associated with the disease development. In addition, we demonstrated that activation of the RAGE/HMGB1 signaling axis in normal keratinocytes induced their proliferation and migration, decreased the sensitivity to drug-induced apoptosis, activated pro-survival molecules, and stimulated intracellular kinase signaling pathways followed by activation of NF-κB and STAT3 transcription factors. Our results are consistent with previous studies which showed that topical application of HMGB1 to skin wounds in mouse models of diabetes enhanced vessel density, accelerated wound healing, and consistently had chemotactic effects on skin fibroblasts and keratinocytes in vitro [24]. It is necessary to stress, however, that RAGE/HMGB1 signaling is not restricted to keratinocytes. HMGB1 expression has been observed by us in normal intestinal mucosa, in chronic rhinosinusitis, and in tumors rich in inflammatory infiltrates such as HNSCC (data not shown). HMGB1 is a chaperone, which has been reported to be present in all nucleated cells and to be expressed in normal and pathological conditions, including inflammation, cancer, and autoimmune disorders [25–27]. Thus, its overexpression and potential involvement in molecular signaling are not specific to cholesteatoma but are manifestations of the inflammatory cascade that HMGB1 sustains via the HMGB1/RAGE axis.

The PI3K/Akt/NF-κB pathway is now recognized as one of the critical pathways in regulating cell survival/apoptosis, migration, and proliferation in pathological conditions such as chronic inflammation or cancer [8, 9, 20]. We demonstrated that triggering of RAGE by HMGB1 activated Akt, induced NF-κB p65 translocation, and resulted in increased proliferation and decreased sensitivity to drug-induced apoptosis in keratinocytes. In agreement with other reports [1, 14, 15], we found that levels of pAkt and pNF-κB molecules were significantly increased and the phosphorylated forms of c-Jun, MEK1, MEK2, MAPKp44/p42 (Erk1/2), and STAT3 were overexpressed in cholesteatoma tissue vs. normal skin epithelium. Furthermore, phosphorylation of all the above-listed molecules was triggered by HMGB1/RAGE interactions in cell lines and correspondingly also in cholesteatoma tissues.

The abnormal behavior of cholesteatoma epithelium seems to be related to the presence of infiltrating immune cells releasing high levels of various cytokines and growth factors. Although IL-1α and TNF-α were not secreted by HaCaT cells, HMGB1 triggered IL-8 production in these cells, confirming previous reports that HMGB1/RAGE interactions can induce IL-8, a cytokine best known for its pro-inflammatory effects on immune cells [27–29]. We suggest, therefore, that RAGE and HMGB1 regulate bone remodeling in chronic inflammation via up-regulation of the IL-8 production, a mediator of bone destruction, in tissue samples of cholesteatoma. The role of RAGE/HMGB1 interactions in bone remodeling has been reviewed [30], and a higher expression of RAGE within cholesteatoma perimatrix, which is adjacent to ossicle surface or to other parts of the temporal bone, might be interpreted as indicative of the RAGE/HMGB1 involvement in this process.

The immune response is critical for the primary defense against middle ear infections. HMGB1 is released extracellularly in response to stress or necrosis and behaves as a cytokine, promoting inflammation [31]. It is possible that keratinocyte proliferation is promoted and sustained by HMGB1 via its interaction with RAGE on keratinocytes. It is also plausible that RAGE/HMGB1 interactions may trigger the signaling pathways involved in the regulation of chronic inflammation as well as bone resorption in cholesteatoma. The cholesteatoma molecular pathogenesis, growth, and inflammation might be related to the co-expression and functional interactions between HMGB1 and RAGE. Their molecular interactions may not be specific to cholesteatoma but, as a prominent part of the inflammatory milieu, are involved in the pathogenesis of this middle ear disease. This suggests that blocking of these interactions might represent a new potentially effective therapy, especially early in disease development. A development of an in vivo animal model of cholesteatoma is crucial for in vivo testing the validity of the proposed mechanisms.

## Electronic supplementary material

Below is the link to the electronic supplementary material.

Supplementary Figure 1 (PDF 141 kb)

Supplementary Figure 2 (PDF 130 kb)
