# Supplementary material for: Molecular signaling of the HMGB1/RAGE axis contributes to cholesteatoma pathogenesis
Source: J Mol Med (Berl). 2014 Nov 12;93(3):305–14. doi: 10.1007/s00109-014-1217-3 (PMC4333301; doi:10.1007/s00109-014-1217-3)
Supplement: Supplementary file 1 — (PDF 141 kb) [file 109_2014_1217_MOESM1_ESM.pdf]

Supplementary Figure 1

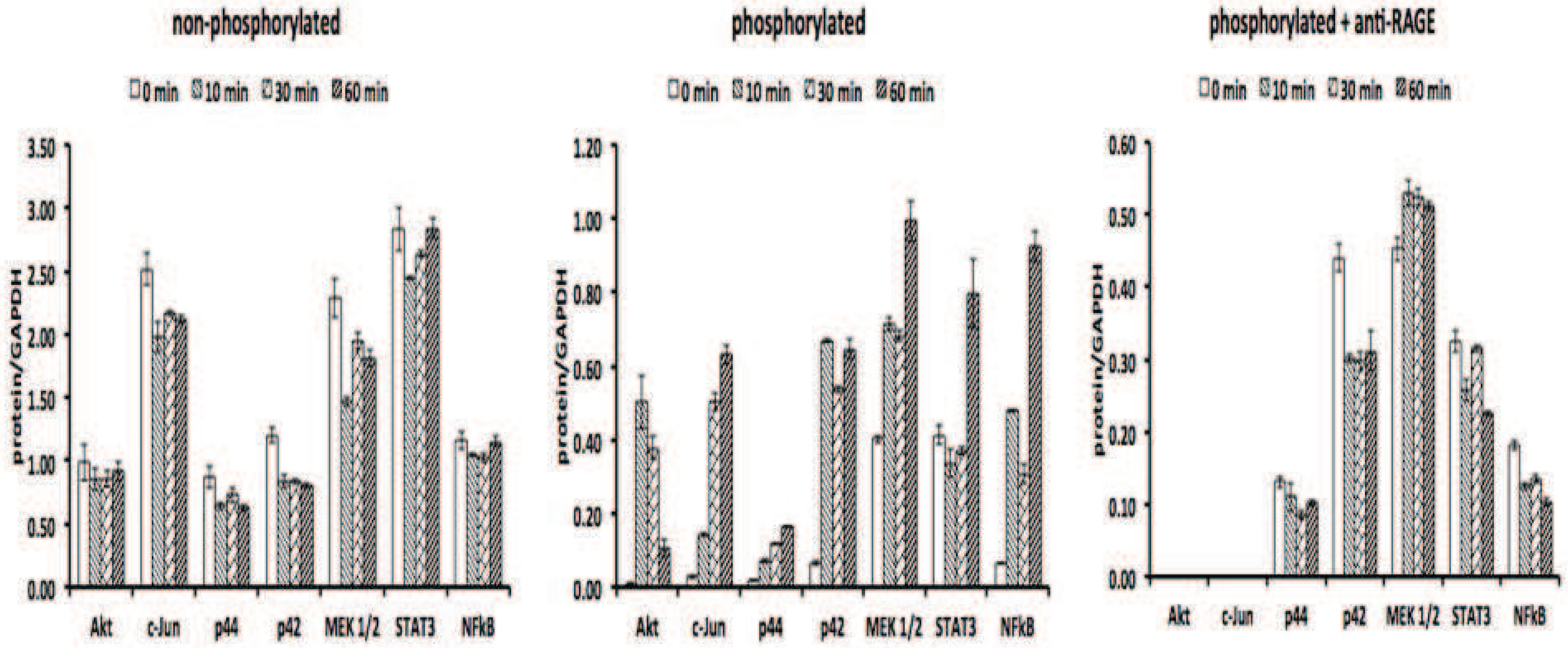

Supplementary Figure 1. The quantification of blots Western blots shown in Figure 5A was performed using ImageJ 1.46r software (National Institutes of Health, USA). The data are means ± SD from 3 individual experiments.
