# Supplementary material for: Molecular signaling of the HMGB1/RAGE axis contributes to cholesteatoma pathogenesis
Source: J Mol Med (Berl). 2014 Nov 12;93(3):305–14. doi: 10.1007/s00109-014-1217-3 (PMC4333301; doi:10.1007/s00109-014-1217-3)
Supplement: Supplementary file 2 — (PDF 130 kb) [file 109_2014_1217_MOESM2_ESM.pdf]

Supplementary Figure 2

- serum free
- control
- +HMGB1
- blocking Ab+HMGB1
- isotype Ab+HMGB1

HaCaT migration

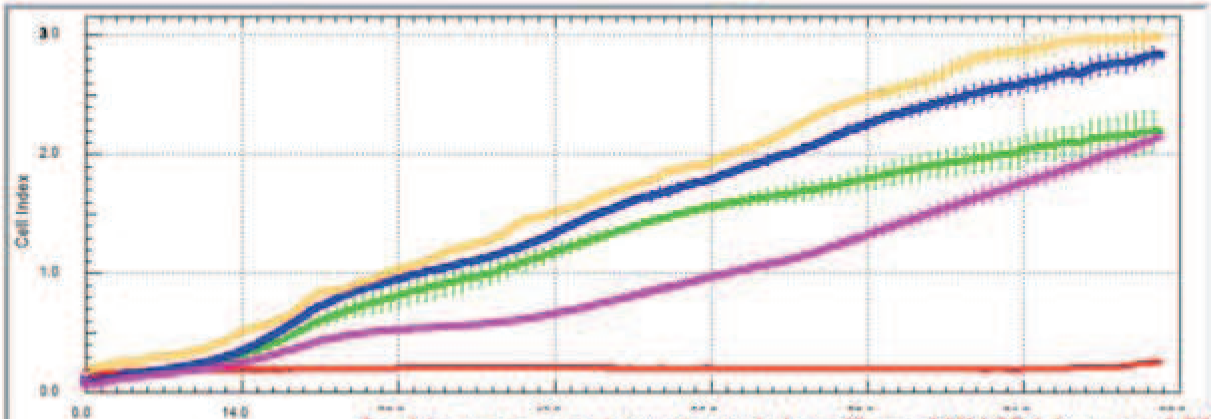

In this paper, we investigated the effect of HMGB1 but not LPS therefore we used in our experiments with cells HMGB1 only.

HaCaT proliferation

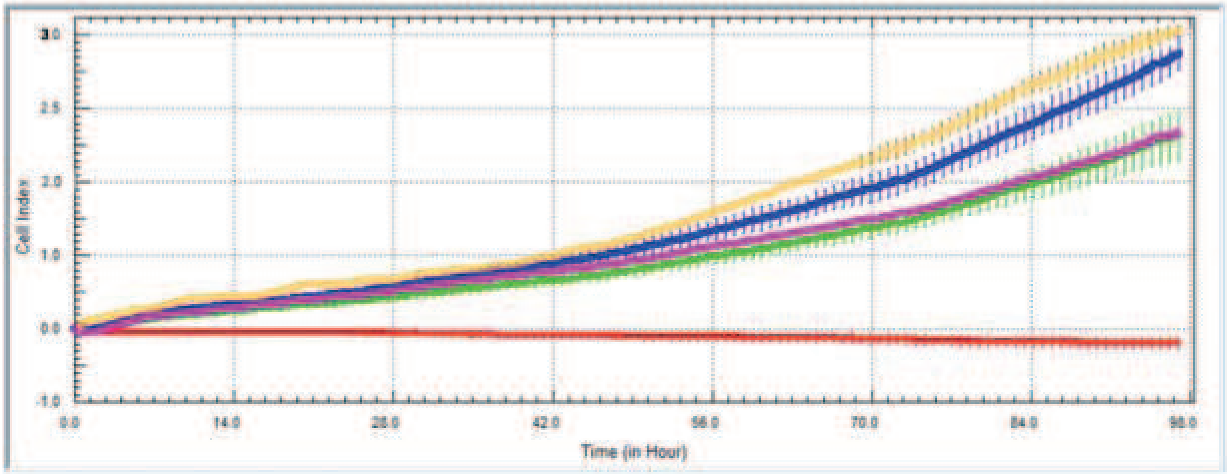

<u>Supplementary Figure 2.</u> Effects of RAGE triggering with HMGB1 (100ng/mL) on TLR4-silenced HaCaT cells proliferation and migration.
